# Differential effects of dietary canola and soybean oil intake on oxidative stress in stroke-prone spontaneously hypertensive rats

**DOI:** 10.1186/1476-511X-10-98

**Published:** 2011-06-13

**Authors:** Annateresa Papazzo, Xavier A Conlan, Louise Lexis, Paul A Lewandowski

**Affiliations:** 1 School of Medicine, Deakin University, Victoria, Australia; 2 Institute for Technology Research and Innovation, Deakin University, Victoria, Australia; 3 School of Human Biosciences, La Trobe University, Victoria, Australia

## Abstract

**Background:**

Canola oil shortens the life span of stroke-prone spontaneously hypertensive (SHRSP) rats compared with rats fed soybean oil when given as the sole dietary lipid source. One possible mechanism leading to the damage and deterioration of organs due to canola oil ingestion is oxidative stress. This study investigated the effect of canola oil intake on oxidative stress in this animal model.

**Method:**

Male SHRSP rats, were fed a defatted control diet containing 10% wt/wt soybean oil or a defatted treatment diet containing 10% wt/wt canola oil, and given water containing 1% NaCl. Blood pressure was measured weekly. Blood was collected prior to beginning the diets and at the end of completion of the study for analysis of red blood cell (RBC) antioxidant enzymes, RBC and plasma malondialdehyde (MDA), plasma 8-isoprostane and plasma lipids.

**Results:**

Canola oil ingestion significantly decreased the life span of SHRSP rats compared with soybean oil, 85.8 ± 1.1 and 98.3 ± 3.4 days, respectively. Systolic blood pressure increased over time with a significant difference between the diets at the 6^th ^week of feeding. Canola oil ingestion significantly reduced RBC superoxide dismutase, glutathione peroxidase and catalase activities, total cholesterol and low-density lipoprotein cholesterol compared with soybean oil. There were no significant differences in RBC MDA concentration between canola oil fed and soybean oil fed rats. In contrast, plasma MDA and 8-isoprostane concentration was significantly lower in the canola oil group compared to the soybean oil group.

**Conclusion:**

In conclusion, canola oil ingestion shortens the life span of SHRSP rats and leads to changes in oxidative status, despite an improvement in the plasma lipids.

## Background

The stroke-prone spontaneously hypertensive (SHRSP) rat is a model of human essential hypertension and stroke, and is derived from the spontaneously hypertensive rat (SHR) and the Wistar-Kyoto (WKY) rat strain [1-4]. In this model, ingestion of canola oil as the sole dietary fat source (added at 10% wt/wt to standard rat chow) has been reported to shorten life span compared to the soybean oil or perilla oil [1,2,5-9]. The sulphur and fatty acid composition of canola oil does not appear to cause the shortened life span [8]. Furthermore, the concentration of phytosterols in canola oil has also been suggested to be a contributing factor. However, conflicting results have shown no clear correlation between the content of phytosterols in the diet and tissues and survival time observed [4,7]. Recent studies suggest that one of the key mechanisms leading to the shortened life span in SHRSP rats is via the acceleration of hypertension-related deterioration of organs [6,7]. An increase in blood pressure [10] and enhanced Na+, K+-ATPase activities [6] have been reported as a result of canola oil ingestion, and may promote the deterioration of organs [6]. Other hypertension-related conditions induced by canola oil ingestion include tissue lesions in the heart and kidney [7].

There is growing evidence that oxidative stress leads to vascular damage and plays a critical role in the pathogenesis of cardiovascular diseases such as hypertension [11,12]. It has been found that production of reactive oxygen species (ROS) is increased in both experimental and clinical hypertension, with a decrease in the antioxidant reserve in vascular tissues [13,14]. In both experimental and clinical hypertension, ROS and oxidative stress have been found to be associated with end-stage organ damage [14]. Hypertension is also a risk factor for stroke [15]. It has been shown that in both ischemic and hemorrhagic stroke, reactive oxygen species (ROS) production is increased, and oxidative stress is a key mediator of tissue damage [15,16].

Cells are protected against ROS by a complex antioxidant system present within the cells, which includes superoxide dismutase (SOD), catalase and glutathione peroxidase (GPx) [17]. Within RBCs, protective mechanisms exist to detoxify and scavenge ROS, and protect endothelial cells against free radical damage [18]. There is an inverse relationship between reduced activities of antioxidants (SOD, GPx and vitamin E) and increased lipid peroxidation products in blood and cardiovascular disease [19]. The activities of the antioxidant enzymes, SOD, catalase and GPx have been found to be reduced both in whole blood and peripheral mononuclear cells in hypertensive subjects [20]. In addition, concentrations of malondialdehyde (MDA) in whole blood and peripheral mononuclear cells have been shown to be increased in hypertensive subjects compared to control [20]. MDA is an end product of lipid peroxidation, and therefore increased concentrations indicate an increase in ROS concentration and resulting oxidative damage [20]. After an acute ischemic stroke the activities of SOD and GPx were reduced and the concentration of MDA was increased in RBCs of human subjects [21]. This study aimed to determine if enhanced oxidative stress and altered antioxidant capacity is associated with canola oil ingestion in SHRSP rats.

## Methods

### Animal husbandry and study design

Experimental design was based on previous life span studies investigating canola oil intake in SHRSP rats [6] to allow direct comparisons to be made. Thirty-four male SHRSP rats (Deakin University, Australia) were used for this study. Ten of the SHRSP rats were sacrificed at 5 weeks of age and blood was collected for analysis (pre-treatment data). Twenty-four, 4 week old SHRSP rats were randomly assigned into two groups of 12 animals each, a control and treatment group, and acclimatized for one week. During acclimatization they were given a standard pellet diet and water ad libitum (Specialty Feeds, Western Australia). Following this, the two groups were fed, respectively, a defatted control diet containing 10% wt/wt soybean oil or a defatted treatment diet containing 10% wt/wt canola oil (Speciality Feeds, Western Australia), and life span was determined. The fatty acid composition and total antioxidant status of the diets are shown in Table 1. Each group was given water containing 1% NaCl to accelerate the development of hypertension. The animals were maintained on a 12 hr light/dark photo-period with a room temperature of 21 ± 2°C. Animal body weights, food intake and water consumption were determined once a week, while the health of the animals was monitored daily. When a rat was found to suffer from stroke, paralysis or to be in pain they were euthanized via intra-peritoneal injection with lethabarb (50 mg/kg) and blood was collected for analysis (post-treatment data). Approval for this project was granted by the Deakin University Animal Welfare Committee.

**Table 1:** Fatty acid composition and total antioxidant status of canola oil and soybean oil diets.

Fatty acid	Soybean oil (%)	Canola oil (%)
14:0 Myristic acid	0.2	0.1
16:0 Palmitic acid	11.0	7.0
16:1 Palmitoleic acid	0.1	0.1
18:0 Stearic acid	4.0	2.0
18:1 Oleic acid	23.0	53.0
18:2 Linoleic acid	48.0	23.0
18:3 Linolenic acid	6.0	10.0
18:4 Stearidonic acid	0	0.5
20:1 Gadoleic acid	0.2	0.1
20:5 EPA	0.2*	0.2*
22:6 DHA	0.5*	0.5*
Total Antioxidant Status (TEAC mmol/L)	0.67 ± 0.01	0.66 ± 0.01

*Fatty acid source coming from fish meal found within the diet.

Information provided by Speciality Feeds (Western Australia), which produced both diets.

### Blood pressure measurement

Blood pressure was measured weekly over the course of their life span using a tail cuff sphygmomanometer (Biopac Systems). For each animal systolic blood pressure was obtained as an average of three readings as each time point.

### Blood collection and processing

After the animal was anaesthetised, blood was collected via cardiac puncture into EDTA coated tubes. Immediately after blood collection, samples were centrifuged at 600 × *g *for 10 minutes at 4°C. The plasma was then removed and stored at -80°C until analysis of plasma lipids: triglycerides, total cholesterol, high density lipoprotein cholesterol (HDL-C) and low density lipoprotein cholesterol (LDL-C), and MDA. RBCs were then washed 3 times by adding an equal volume of 0.9% (w/v) NaCl, mixed carefully and centrifuged at 600 × *g *for 10 minutes at 4°C. The supernatant was removed and discarded. An equal volume of cold distilled water and RBCs were mixed well to lyse the cells. The hemolysate was stored at - 80°C for subsequent analysis of antioxidant enzymes: SOD, catalase and GPx, and MDA.

### Erythrocyte antioxidant enzymes

Superoxide dismutase (SOD) activity was determined using a commercially available kit (Cayman Chemical Company) following the manufacturer's instructions. This assay utilizes xanthine oxidase and hypoxanthine to generate superoxide radicals that are detected by tetrazolium salt with absorbance read at 540 nm using a microplate analyser (Fusion-Alpha HT, PerkinElmer). One unit of SOD is defined as the amount of enzyme required to inhibit the distmutation of the superoxide radical by 50%.

Catalase activity was determined using a commercially available kit (Cayman Chemical Company) following manufacturer's instructions. This method is based on the reaction of methanol with the enzyme in the presence of an optimal concentration of hydrogen peroxide. The absorbance was read at 540 nm using a microplate analyser (Fusion-Alpha HT, PerkinElmer).

GPx activity was determined using a modification of the method of Wheeler et al. (1990) [22]. This assay is based on the oxidation of nicotinamide adenine dinucleotide phosphate (NADPH) following the reduction of t-butyl hydroperoxide. A decrease in absorbance at 340 nm results from oxidation of NADPH to NADP+ and the rate of this decrease is proportional to the GPx activity in the sample.

All erythrocyte enzyme activities were normalised to haemoglobin concentration, which was determined by adding 20 μl of 200/1 hemolysate and 480 μl of Darbkin's reagent. The sample was left to stand at room temperature for 5 minutes and the absorbance read at 540 nm using a spectrophotometer (Biochrom).

### Lipid peroxidation

MDA in plasma and erythrocytes was determined via high performance liquid chromatography (HPLC) according to the method by Sim et al. (2003) [23]. Briefly, 100 μl hemolysate or 50 μl plasma samples were hydrolysed with 1.3 M sodium hydroxide, incubated at 60°C for 60 minutes and cooled on ice for 5 minutes. To precipitate the proteins, 35% perchloric acid was added, cooled on ice for 5 minutes and centrifuged at 3500 × g for 10 minutes. The samples were protected from light from this step onwards. To the supernant 30 μl of 2,3-dinitrophenylhydrazine reagent was added and incubated for 10 minutes at room temperature. The aqueous phase was extracted twice with hexane and evaporated. The dry extract was reconstituted with 100 μl mobile phase, and a 45 μl injection volume was used. MDA concentrations were determined at 310 nm using HPLC (Agilent Technologies) with an Eclipse XDB-C18 column (150 × 4.6 mm, 5 μm, 1 ml/min flow rate, 9.8 MPa backpressure). External standards (5, 10, 20 and 40 μM) of MDA aliquots of suitable concentrations were used.

Total 8-isoprostane concentrations were analysed in plasma using an enzyme immunoassay (EIA) kit (Cayman Chemicals) following manufactures instructions. Prior to analysis plasma samples were hydrolysed by addition of 25 μl 2 M NaOH to each 100 μl plasma sample. The samples were incubated at 45°C for 2 hours. Following this, 25 μl 10N HCl acid was added and the samples were centrifuged for 5 minutes at 12,000 × *g*. The supernatant was removed and used for the determination of total 8-isoprostane using the EIA kit. This assay is based on the competition between 8-isoprostane and an 8-isoprostane acetycholinesterase (AChE) conjugate for a limited number of 8-iso-PGF2α-specific rabbit anti-serum binding sites, values were expressed as pg/ml of plasma.

### Plasma lipids

Plasma triglycerides, total cholesterol and High density lipoprotein cholesterol (HDL-C) were determined using commercially available kits (Thermo Electron Corporation) in a 96 well plate format (Fusion-Alpha HT, PerkinElmer), following the manufactures instructions.

Low density lipoprotein cholesterol (LDL-C) was determined using the Friedewald equation [24]: LDL cholesterol = Total Cholesterol - HDL cholesterol - (TG/5).

### Statistical analysis

Statistical analysis was performed using the SPSS statistical package (version 17.0, SPSS Inc.) for repeated measures ANOVA and one-way ANOVA. The results are represented as mean ± SEM. Comparisons between groups for animal body weight, food intake and water intake data were analysed using repeated measures ANOVA. A post hoc pair-wise comparison was also carried out. Statistical analysis of the survival time data was performed by Log-Rank and Wilcoxon's nonparametric tests using GraphPad Prism 5 software. The differences between group means for the antioxidant enzymes, MDA and plasma lipids were made by one-way ANOVA. Significance was established at the 95% confidence level (*P *< 0.05).

## Results

### Establishment of canola-induced life shortening model

**Figure 1:**
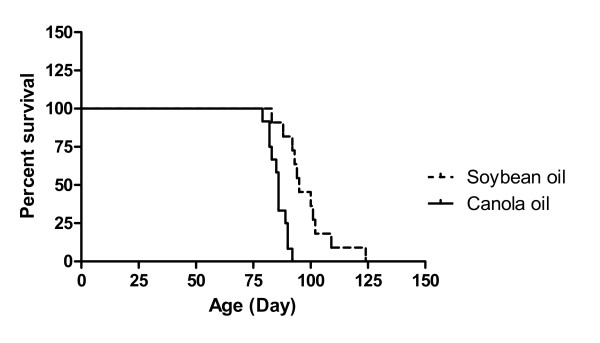
**Survival curves of SHRSP rats fed a diet containing 10% soybean oil or canola oil**. The curves are significantly different (*P *< 0.001, Log-rank and Wilcoxon's test). At the commencement of the study the number of animals in each group was 12.

Canola oil ingestion significantly decreased the life span of SHRSP rats (*P *< 0.001). Mean lifespan of the two diet groups was 98.3 ± 3.4 days in the soybean oil group compared to 85.8 ± 1.1 days in the canola oil group (Figure 1), confirming the establishment of the model.

### Body weight, food intake and water intake

**Figure 2:**
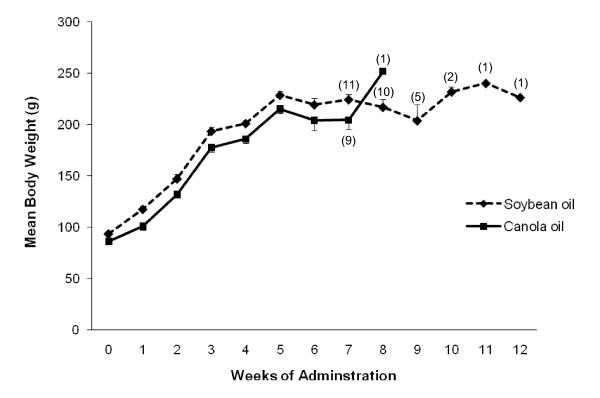
**Mean body weight of SHRSP rats fed canola oil compared with soybean oil diet**. Vaules are means ± SEM. At the commencement of the study the number of animals in each group was 12. The numbers on the graph repesent the n in each group. *P < 0.05 represents a significant difference between canola and soybean oil groups.

**Figure 3:**
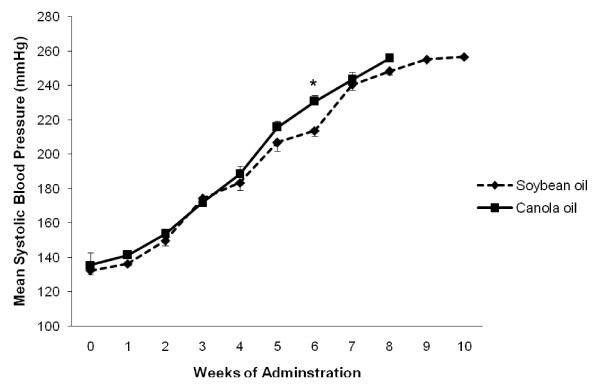
**Food and water intake of SHRSP rats fed canola oil compared with soybean oil diet**. Values are means ± SEM. At the commencement of the study the number of animals in each group was 12. The numbers on the graph repesent the n in each group.

Body weight of the animals increased gradually until the 5^th ^week of administration in both diet groups. There was no significant difference between soybean oil and canola oil groups (Figure 2). There was also no significant difference in food consumption and water intake between the soybean oil and canola oil groups (Figure 3).

### Blood pressure

**Figure 4:**
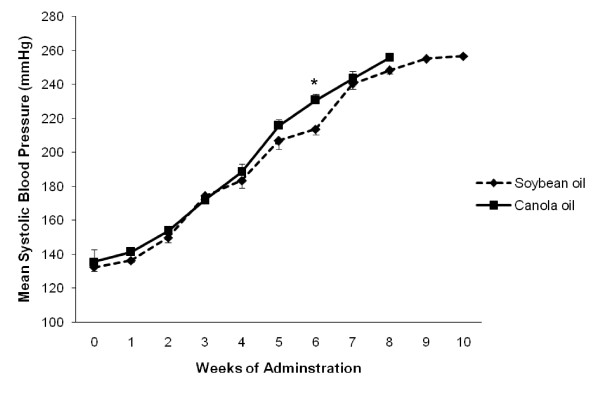
**Mean systolic blood pressure of SHRSP rats fed canola oil compared with soybean oil diets**. Vaules are means ± SEM. At the commencement of the study the number of animals in each group was 12. *P < 0.05 represents a significant difference between canola and soybean oil groups.

Mean systolic blood pressure increased over time in the soybean oil and canola oil groups. At the 6^th ^week of administration the mean systolic blood pressure was significantly higher in the canola oil group compared to soybean oil, 230.7 ± 3.5 and 213.6 ± 3.3 mmHg, respectively (*P *< 0.05, Figure 4).

### Antioxidant enzymes and oxidative damage

Markers of antioxidant status and oxidative damage are represented in Table 2. Canola oil ingestion significantly reduced (*P *< 0.05) the activities of RBC SOD, GPx and catalase compared to soybean oil. The activities of RBC SOD and GPx were significantly lower (*P *< 0.05) in the canola oil group compared to pre-treatment, however, there were no significant differences between the soybean oil group and pre-treatment.

**Table 2:** Antioxidant status and oxidative damage in SHRSP rats fed canola oil compared with soybean oil diets.

	Pre-treatment	Post-treatment	
		
		Soybean oil	Canola oil
RBC SOD (U/gm Hb)	1608.7 ± 121.8	1454 ± 111.3	1087.2 ± 92.9*^, #^
RBC GPx (U/gm Hb)	523.0 ± 25.9	512 ± 36.6	415 ± 19.4*^, #^
RBC Catalase (mmol/min/gm Hb)	155.6 ± 13.4	208.2 ± 35.1	142.4 ± 15.9*
RBC MDA (μM)	28.6 ± 1.6	16.6 ± 3.5^#^	21.8 ± 3.9
Plasma MDA (μM)	11.8 ± 0.5	17.9 ± 0.5^#^	14.9 ± 1.3*^, #^
Plasma 8-isoprostane (pg/ml)	75.2 ± 12.3	103.2 ± 7.6^#^	83.2 ± 5.6*

Values are means ± SEM; Number of animals at post-treatment was 10. Number of animals in each group at post-treatment for soybean oil and canola oil was 9 and 8, respectively. SOD, superoxide dismutase; GPx, glutathione peroxidase; MDA, malondialdehyde.

*P < 0.05 represents a significant difference between canola and soybean oil groups;

^#^*P *< 0.05 represents a significant difference between pre-treatment vs post-treatment.

Canola oil ingestion significantly decreased (*P *< 0.05) plasma MDA and 8-isoprostane in SHRSP rats compared to the soybean oil group. Nevertheless, plasma 8-isoprostane in the soybean oil group and MDA in both canola oil and soybean oil groups was significantly higher (*P *< 0.05) than the pre-treatment group. Lastly, RBC MDA in the soybean oil group was significantly lower (*P *< 0.05) than pre-treatment.

### Plasma lipids

Canola oil ingestion significantly reduced (*P *< 0.05) the concentration of total cholesterol and LDL-C compared with soybean oil. Total cholesterol and LDL-C were significantly lower (*P *< 0.05) in the canola oil group compared with pre-treatment, however, there were no significant differences between the soybean oil group and pre-treatment. HDL-C in both canola oil and soybean oil groups was significantly lower (*P *< 0.05) than pre-treatment. There were no significant differences (*P *> 0.05) in the concentration of triglycerides between groups (Table 3).

**Table 3:** Plasma lipids in SHRSP rats fed canola oil compared with soybean oil diets.

	Pre-treatment (mmol/L)	Post-treatment (mmol/L)	
		
		Soybean oil	Canola oil
Total cholesterol	7.9 ± 0.2	7.9 ± 0.4	6.67 ± 0.3*^, #^
LDL-C	5.3 ± 0.2	5.6 ± 0.4	4.57 ± 0.2*^, #^
HDL-C	2.4 ± 0.1	1.8 ± 0.1^#^	1.67 ± 0.1^#^
Triglycerides	1.4 ± 0.1	1.4 ± 0.1	1.21 ± 0.1

Values are means ± SEM; Number of animals at post-treatment was 10. Number of animals in each group at post-treatment for soybean oil and canola oil was 9 and 8, respectively. SOD, superoxide dismutase; GPx, glutathione peroxidase; MDA, malondialdehyde.

*P < 0.05 represents a significant difference between canola and soybean oil groups;

^#^*P *< 0.05 represents a significant difference between pre-treatment vs post-treatment.

## Discussion

Previous studies have shown that the life span of SHRSP rats is shortened when canola oil is the only dietary fat source [1,2,5-8] which is confirmed by the present study, which showed shortened life span of approximately 13% with canola oil feeding compared with soybean oil under 1% NaCl loading. Our results are similar to the study published by Ratnayake et al. (2000b) [9], which produced a 13% reduction in life span with canola oil feeding. Canola oil has been reported to shorten the life span of SHRSP rats compared with soybean oil, 254 ± 12 and 416 ± 16 days, respectively, even without NaCl loading [1].

Evidence indicates that canola oil intake has an effect on blood pressure in the SHRSP rat and its related strains. However, blood pressure in the present study is not a key contributing factor to the shortened life span, as there was only one time difference between soybean oil and canola oil. The results of the present study show an increase in systolic blood pressure over time in both treatment groups, with a difference between the groups at the 6^th ^week of administration. A study by Huang et al. (1996) observed no significant change in systolic blood pressure in the canola oil group compared to the soybean oil group at 4 and 8 weeks of age [5]. Another study by Ratnayake et al. (2000a) found no significant differences in the SBP among different dietary groups in SHRSP rats [8]. While previous literature has shown that decreased antioxidants and oxidative stress play a role in hypertension, to our knowledge this is the first study to investigate the effect of canola oil on measures of antioxidant status and oxidative damage in SHRSP rats. In the present study canola oil ingestion decreased the activities of SOD, GPx and catalase compared with the soybean oil group by 25%, 18% and 31%, respectively. Previous studies have analysed several antioxidant enzymes in SHR and WKY rats. In the SHR, the activity of RBC GPx and the activities of SOD and catalase in the hepatic cytosol were found to be reduced [25]. In WKY rats, canola oil ingestion increased the activity of SOD and reduced the activity of catalase in the hepatic cytosol [26], while an earlier study showed reduced activities of SOD and catalase in the hepatic cytosol of WKY rats [27]. Our data are largely consistent with these findings. Taken together these results indicate that canola oil ingestion affects antioxidant enzyme activity in different tissues. Indeed, previous research has shown an inverse relationship between erythrocyte GPx activity and the incidence of cardiovascular disease [28]. Given the physiological significance of the decreased erythrocyte GPx activity, further research is required to determine the mechanism to explain the canola-oil induced changes to antioxidant enzyme activity.

In the present study, canola oil ingestion decreased the plasma 8-isoprostane and MDA concentration when compared to the soybean oil group. These results are unexpected given the canola oil-induced decrease in erythrocyte antioxidant enzyme activity. The mechanism to explain the decreased plasma 8-isoprostane and MDA concentration despite a decrease in RBC antioxidant enzyme activity is currently unknown. Plasma 8-isoprostane in the soybean oil and MDA in both the canola oil and soybean oil groups was higher than the pre-treatment group. In a previous study in WKY rats, canola oil ingestion increased lipid peroxide levels in the hepatic cytosol [26], while an earlier study showed no change in lipid peroxide levels in the hepatic cytosol of SHR [25]. The increased plasma 8-isoprostane in the soybean oil group and MDA concentrations in both dietary groups compared to pre-treatment indicates an increased amount of ROS induced lipid peroxidation over time independent from the consumption of either canola or soybean oil. RBC MDA was decreased in the soybean oil group compared to pre-treatment, and indicates a decrease in erythrocyte lipid peroxidation over time. Previous investigations that have determined the potential for cellular membranes to undergo oxidation based on their fatty acid composition [29] suggest that the change in lipid peroxidation observed in the present study is in contrast to what may be expected based on the fatty acid composition of canola and soybean oil. Canola oil has relatively more n-9 monousnsatruated fatty acids, more readily oxidised, compared to soil bean oil that has proportionally more n-6 polyunsaturated fatty acids, less readily oxidised [4]. However only focusing on the fatty acid composition alone of the two oils is too simplistic as the TAS for both diets was the same and the lower concentration of RBC MDA in the soybean oil group may have resulted from the higher RBC antioxidant activities compared to the canola oil group. Whichever the case the specific mechanism for these differences in RBC lipid peroxidation associated with the consumption of canola or soybean oil was not identified in the present study. Therefore, more research is clearly required to investigate the effect of canola oil intake on oxidative damage in SHRSP rats.

Canola oil ingestion reduced the concentration of total cholesterol and LDL-C compared with soybean oil and the pre-treatment group. HDL-C was lower in both dietary groups compared with pre-treatment, and there were no changes found in the triglyceride levels between the groups. However, previous studies in SHR and WKY rats have shown increases in total cholesterol, triglycerides and HDL-C with administration of canola oil compared to soybean oil [25-27]. A study by Ohara et al. (2006) found no changes in the plasma lipids in the canola oil group after an eight week feeding trial in SHRSP rats [7]. Canola oil is considered to provide protective cardiovascular effects due to its favourable fatty acid composition [8]. Canola oil has a high content of oleic acid (55-60%), a low content of saturated fatty acids (6-7%), and provides a good source of omega-3 fats [4]. The present study supports the beneficial health effects of canola oil, as the plasma lipids were reduced in SHRSP rats. A Previous study has shown that a canola oil rich diet reduced plasma cholesterol, LDL-C and HDL-C concentrations in middle-aged and elderly hypercholesterolemic subjects [30]. Canola oil has also been shown to reduce total plasma cholesterol and LDL-C in the young men [31]. Whilst this present study supports the beneficial health effects of canola oil on blood lipids, it should also be pointed out that the metabolism of cholesterol in rats is different from cholesterol metabolism in humans [32]. Therefore direct extrapolation of our circulating cholesterol results into humans must be done so with caution. However, the effects of canola oil ingestion in the SHRSP rat is still detrimental as this oil leads to a shorten life span in SHRSP rats.

There are a number of limitations associated with the present study in addition to the differences between cholesterol metabolism in rats and humans. The first is the question of whether the life shortening effect associated with canola oil consumption is specific to rats originally derived from the normotensive Wistar Kyoto (WKY) strain. When canola oil was the only dietary fat fed to WKY rats, a significant increase in systolic blood pressure after five weeks of canola oil feeding was observed and persisted until the conclusion of the trial at week 13, compared to soybean oil fed rats [27]. In addition when spontaneously hypertensive rats (SHR) rats were fed canola oil canola they developed vascular lesions in the kidney, had elevated plasma lipids and glucose-6-phosphate dehydrogenase (G6PD) activation in the liver and erythrocytes compared to SHR fed soy oil [25]. In human populations there is only one published incidence of canola oil being associated with the development of disease [33] and this due was to manufacturing error when producing canola oil. The present study did not directly investigate the effect of canola oil on cell membrane fatty acid composition, fluidity, antioxidant capacity or oxidized membrane lipids. Although MDA was measured in plasma and lysed red blood cells, the membrane fraction of red blood cells or other organs was not isolated prior to measurement of MDA. A change to cell membrane composition has previously been investigated as a potential mechanism to explain the life shortening effects of canola oil in the SHRSP. These previous studies suggested that hyperabsorption and accumulation of plant sterols found in canola oil altered the fluidity of the membranes present in red blood cells, liver and kidney and contribute to the life shortening effect observed in SHRSP rats [9]. Finally NaCl loading is used to accelerate the development of hypertension in the SHRSP [1,2,5-8], however it may be masking the effects of canola oil in the SHRSP rat, and thus further investigation on the life shortening effect of canola oil in the absence of NaCl are required.

In conclusion, we have shown that canola oil ingestion mediated life span shortening of SHRSP rats leads to changes in oxidative status. The plasma lipids were reduced after canola oil ingestion highlighting the health benefits of canola oil intake. Despite the improvement in the plasma lipids, canola oil was detrimental to the SHRSP rat as their life span is reduced. Further research is required to determine whether oxidative stress plays a role in SHRSP rats due to canola oil ingestion.

## Competing interests

The authors declare that they have no competing interests.

## Authors' contributions

AP participated in the design of the study, carried out the analysis and interpretation of data and drafted the manuscript. XAC helped with the MDA analysis. LL contributed to the interpretation of data and revised the manuscript. PAL participated in the design of the study, contributed to the interpretation of data and revised the manuscript. All authors read and approved the final manuscript.
